# Effect and Challenges of an Integrated Nutrition-Intervention Package Utilization among Pregnant Women and Lactating Mothers in Rwanda: An Exploratory Qualitative Study

**DOI:** 10.1016/j.cdnut.2022.100018

**Published:** 2022-12-23

**Authors:** Michael Habtu, Alemayehu Gebremariam Agena, Maryse Umugwaneza, Monica Mochama, Cyprien Munyanshongore

**Affiliations:** 1 School of Public Heatlh, College of Medicine and Health Sciences, University of Rwanda, Kigali, Rwanda; 2 Catholic Relief Services of Rwanda, Kigali, Rwanda; 3 Department of Public Heatlh, School of Health Sciences, Mount Kenya University, Thika, Kenya

**Keywords:** challenges, implementation, integrated nutrition intervention, nutrition-specific, nutrition-sensitive interventions

## Abstract

**Background:**

Malnutrition among pregnant women and lactating mothers remains an issue of public health concern in developing countries. The *Gikuriro* program, an integrated nutrition-specific and nutrition-sensitive intervention, was implemented in 5 districts of Rwanda for 5 y to address this problem. Postprogram quasi-experiments showed significant effect of the intervention on maternal and child undernutrition. Notwithstanding, there was a need for a qualitative study to explore the views of the beneficiaries and implementers regarding its benefits, challenges, and limitations to inform future interventions.

**Objective:**

This study aimed to explore the effect and challenges of an integrated nutrition-intervention program among pregnant women and lactating mothers.

**Methods:**

This was a qualitative study involving 25 community health officers and 27 nutritionists as key informants and 80 beneficiaries in 10 focus group discussions. All interviews and group discussions were audio-recorded, transcribed verbatim, translated into English, and double coded. A deductive and inductive content analysis approach was used with the help of ATLAS.ti, version 9.15.

**Results:**

The study identified several positive effects, such as improved knowledge and skills on nutrition, a positive mindset toward a balanced diet, perceived improved nutrition, and economic independence among pregnant women and lactating mothers. However, some of the main obstacles of the integrated nutrition intervention were lack of awareness of the program, negative beliefs, poverty, lack of spousal support, and time constraints. Moreover, the study identified a main limitation: the lack of inclusiveness for all social categories.

**Conclusions:**

This study demonstrates that integrated nutrition interventions have perceived positive effect on nutrition; however, such interventions may face some challenges and limitations. These findings suggest that, apart from contributing to the body of evidence for scale up of such interventions in resource-limited settings, economic challenges and misconceptions have to be addressed to maximize the effect of such interventions.

## Introduction

Maternal undernutrition during pregnancy and after delivery remains a dire public health problem. It affects the well-being of mothers and their offspring and has a long-lasting effect on the survival, growth, and development of children during the first 1000 d and beyond [1, 2]. It is worth noting that Rwanda made notable improvements in the nutritional status of children over the past decade. According to Rwanda Demographic and Health Survey, the prevalence of stunting among children fell from 44% in 2010 to 38% in 2014–2015 and 33.1% in 2019–2020, whereas underweight declined from 18% in 2005 to 9% in 2014–2015 and a slight decline (8%) in 2019–2020 [[3], [4], [5], [6]]. These improvements could be attributed to the high-level political commitment, multisectoral coordination efforts, and aggressive interventions of comprehensive nutrition programs by the government and its development partners. However, further progress is needed, especially on maternal and neonatal nutritional status, because the trend of maternal anemia and underweight and birth weight according to the demographic survey remain the same in the last decade [6].

Community-based intervention strategies using locally accessible personnel and resources to deliver key maternal and neonatal health and nutrition interventions are now widely recognized [7, 8]. To achieve long-term effect of improved nutrition, implementing an integrated multisectorial approach is important [9]. In light of this, *The Lancet* series for *Maternal and Child Nutrition* in 2013 proposed nutrition-specific and nutrition-sensitive interventions to improve nutrition and for optimum fetal and child growth and development [7, 10]. Nutrition-specific interventions are interventions that address the immediate determinants of maternal, fetal, and child malnutrition, such as deprived health and insufficient dietary intake. On the contrary, the nutrition-sensitive program addresses the underlying and basic determinants of maternal, fetal, and child malnutrition by improving agricultural productivity, food security, social safety nets, women empowerment, early child development, and water, hygiene, and sanitation (WASH) [7, 10, 11]. Moreover, nutrition-sensitive interventions are believed to serve as delivery platforms for nutrition-specific interventions, potentially increasing their scale, coverage, and effectiveness [9, 10].

Considering that there is still high maternal and child undernutrition, the Government of Rwanda and its development partners implemented evidence-based nutrition-specific and nutrition-sensitive intervention packages identified by *The Lancet Maternal and Child Nutrition* series [7, 10] through a project called *Gikuriro* (good growth as opposed to stunting). The project was funded by the US Agency for International Development (USAID), Rwanda. It was implemented by Catholic Relief Services (CRS) along with the Netherlands Development Organization and 6 local nongovernmental organization partners between November 2015 and November 2020 in 5 districts. The overall goal of the program was to improve the nutritional status of women of reproductive age and children aged <5 y, with an emphasis on the 1000-d window of opportunity from pregnancy until a child’s second birthday.

Recent quantitative studies have been performed to evaluate the effect of the *Gikuriro* program on maternal nutritional status and birth weight. Both postprogram quasi-experiments revealed that the intervention led to significant reduction of low birth weight and maternal undernutrition [12, 13]. The main success factor having been attributed to the integration of nutrition-specific and nutrition-sensitive intervention package. However, there are various demand and supply barriers that limit pregnant women and lactating mothers from using integrated nutrition interventions. These include maternal factors (e.g., lack of awareness/knowledge, low educational status, poor dietary habits, and dependency syndrome); household factors (e.g., heavy workload, poor husband support, and lack of economic resources); community factors (e.g., food taboos and avoidances, sociocultural and religious influences, selling of food products, and limited sources of nutrition information); and health/nutrition service factors (e.g., poor access to health facilities, poorly equipped health facilities, focus only on child health and nutrition, and poor coordination among nutrition-specific and nutrition-sensitive sectors) [14, 15].

Therefore, this study explored the perceived benefits, challenges, and limitations among beneficiaries (pregnant women and lactating mothers) and implementers of the program to inform future integrated nutrition interventions to improve their delivery. The findings of this study would further provide lessons on how the success of this program can be replicated.

## Methods

### Study design and setting

An exploratory qualitative approach was conducted to explore the effect, challenges, and limitations to the utilization of integrated nutrition-specific and nutrition-sensitive intervention package (*Gikuriro* program) among pregnant women and lactating mothers. The study was performed in 5 districts where the *Gikuriro* program was implemented by CRS in close collaboration with the Government of Rwanda and other partners. The targeted districts were Kayonza and Ngoma from Eastern Province, Nyabihu from Western Province, and Kicukiro and Nyarugenge from Kigali City. These districts were selected in 2015 owing to high proportion of malnutrition among children and a limited number of local development partners working on health and nutrition of children and reproductive age women. The study was conducted between March and June 2021.

### Description of the integrated nutrition-intervention package

The *Gikuriro* program combined 4 nutrition interventions: 1 nutrition-sensitive and 3 nutrition-specific interventions, to form an integrated nutrition-intervention package. The nutrition-specific intervention was nutrition education and counseling, whereas the 3 nutrition-sensitive components were promotion of agricultural productivity, promotion of financial literacy/economic resilience, and improved access to WASH services. The intervention took place between 2015 and 2020. It was funded by USAID and implemented by CRS in partnership with the Government of Rwanda, Netherlands Development Organization, and other Rwandan civil-society organizations.

#### Description of the nutrition-specific intervention

The women received nutrition education and counseling by community health workers (CHWs) and nutritionists. First, the nutritionists and CHWs in charge received training on the counseling guide module. Then, the CHWs in charge in turn trained the CHWs at the village level. The trained nutritionists counseled the pregnant women about nutrition during regular antenatal care visits, and sessions lasted about 30 to 45 min each. Moreover, the nutritionists trained the women through cooking demonstrations and about balanced diet through Village Nutrition School. Participants were required to come along with food items for the demonstration. In addition, the CHWs supervised by community health officers (CHOs) gave further nutrition education and counseling at the household level. Furthermore, the CHWs received in-service training monthly.

#### Description of the nutrition-sensitive interventions

In this intervention, 3 components were implemented: promotion of increased agricultural productivity, promotion of financial literacy/economic strengthening, and improved access to WASH services.

*Increased agricultural productivity*. Beneficiaries were taught to practice nutrition-sensitive agriculture and to increase agriculture production using of bio-intensive agriculture techniques. They were grouped into farmer field learning school and advised on how to improve their production to attain food security at household level.

*Promotion of financial literacy/economic strengthening*. The economic status of the women was improved by grouping them into Saving and Internal Lending Communities (SILC) as a way of responding to financial problems that prevent them from attaining better nutrition. The main methods of SILC were training those in charge of economic strengthening and project coordinators and district cooperative staff; then, the trained staff train the sector cooperative officers where they in turn train field agents from community. Furthermore, the field agents sensitize the people about SILC and form the groups. The goal was to help these women better manage their existing resources by teaching them basic financial management skills. This enabled the poor to build up useful lump sums without incurring excessive debt or interest charges.

*WASH interventions*. Improved WASH services were implemented using Community-Based Environmental Health Promotion Program approach through community health clubs (CHCs) at the village level. Community-Based Environmental Health Promotion Program is a hygiene behavior change approach to reach communities and empower them to identify their personal and domestic hygiene needs. CHC and a demonstration site at every village was formed and initiated. The CHCs were responsible for ensuring that levels of hygiene were monitored, together with the CHW facilitator, who visited each household to observe the household sanitation and environmental conditions.

### Study participants

The target population comprised pregnant women and lactating mothers who were beneficiaries of the *Gikuriro* program from the 5 districts. Ten focus group discussions (FGDs), 2 from each district, were conducted among the pregnant women and lactating mothers. A total of 80 women (40 pregnant women and 40 lactating mothers) participated in the group discussions. Each FGD comprised 4 pregnant women and 4 lactating mothers. They were selected with the help of CHOs (supervisors of CHWs) who had the records of the participants, which assisted researchers to select participants who fulfilled the inclusion criteria. The women were selected based on their participation in the *Gikuriro* program and educational background. Only women who had been in the program for its full duration were included. Moreover, in each district, one FGD constituted women with postprimary education and the other FGD women with lower than secondary level education. The discussions were conducted at the health centers and round transport for the participants was covered.

In addition, key informant interviews (KIIs) were conducted among CHOs (*n* = 25) and nutritionists (*n* = 27) actively involved in the implementation of the project. One CHO and 1 nutritionist were selected from each health facility in the 5 districts. There was only 1 CHO and 1 nutritionist assigned to each health facility (health centers and district hospitals). However, those newly assigned to the post and those with <1 y of experience with the *Gikuriro* program were excluded.

### Data collection tool and procedure

Pretested modified FGD and KII guides were used to collect data based on the demand-side and supply-side barriers [14]. Demand-side barriers refer to the individual, household, or community factors that influence the demand for utilization of nutrition-intervention packages, whereas supply-side barriers refer to the characteristics of the intervention, such as quality, accessibility, and availability. The guides were translated into Kinyarwanda (the local dialect).

The discussions and interviews were conducted by 2 women (one with Master’s degree in public health and the other with a Master’s in nursing). Both were highly experienced as they had previously conducted several qualitative data collections. However, they were trained by the principal investigator regarding the objectives of the study, the guide questions, approach, recording, and confidentiality. Kinyarwanda was used to collect the data using a face-to-face approach. The FGDs and KIIs were audio-recorded where there was optimal privacy and low noise in the respective selected health centers. Discussions were held in a circle to facilitate face-to-face dialog and numbers from 1 to 8 were assigned to participants to allow anonymous transcription. A moderator and a notetaker were assigned to each FGD, whereas, for KIIs, only 1 person was assigned for notetaking and interviewing. At the end of the FGDs and KIIs, the keynotes were shared/reviewed to validate what they had said. The audio-recorded data were saved on a personal computer for security and confidentiality. On average, the FGD lasted ∼1 h and the interview ∼45 min.

### Data analysis plan

The discussions and interviews were first transcribed verbatim in Kinyarwanda and translated into English. These transcriptions and translations were supervised and double checked to enhance validity. The translated data were saved using unique names and imported to ATLAS.ti version 9.15 (Atkas.ti) for coding and analysis. Four main stages—decontextualization, recontextualization, categorization, and compilation [16]—were followed. Decontextualization was used to familiarize and get sense of the transcriptions through reading and re-reading. In this regard, coding was using with both a deductive approach using the codebook developed according to the study objectives and an inductive approach to allow for emerging themes during assigning quotations. There were 2 coders, and an agreement was reached through continuous discussion between them. Induction coding was performed independently. The second process of recontextualization was used to make sure that the original text and list of the final codes were covered/addressed according to the study objectives. Through categorization, similar thematic content was sorted and classified into subthemes according to the subtheme categories (codes). Finally, the categories were interpreted and descriptive quotes representing key themes were compiled for the final report.

### Ethics statement

The study was reviewed and received ethical approval from the institutional review board of the University of Rwanda, College of Medicine, and Health Sciences (No. 362/CMHS IRB/2020), in compliance with the tenets of the Helsinki Declaration. Subsequently, the study received approval to collect data from the Ministry of Health’s National Health Research Committee (No. NHRC/2020/PROT/046). Before data collection, each participant was informed about the purpose, procedure, voluntary participation, and confidentiality of the study. A verbal audiotaped consent was obtained from each participant. Participants did not receive any compensation apart from the round trip transportation cost. From the start of recording, participants used an assigned number, and no identifiers were recorded. The principal investigator was responsible for the security of the data (transcripts). There were no direct benefits for participation.

## Results

### Overview of core and subthemes

Table 1 provides the core themes, subthemes, and categories for the FGDs and KIIs. The core themes were effect/benefits, challenges, and limitations of using the *Gikuriro* program (integrated nutrition-specific and nutrition-sensitive intervention package). The key subthemes identified for effect were as follows: *1*) enhanced knowledge and skills on nutrition, *2*) perceived improved nutritional status, and *3*) women empowerment/independence. The following 4 subthemes were identified as key challenges in the utilization of this integrated nutrition-specific and nutrition-sensitive intervention: *1*) lack of awareness, *2*) negative attitude/beliefs, *3*) poverty/economic constraints, and *4*) lack of husband support and time constraints. Finally, 1 key limitation of the intervention emerged during the analysis, which is the lack of inclusiveness to all social groups.

**TABLE 1:** Core themes, subthemes, and subtheme categories

Main themes	Subthemes	Subtheme categories (codes)
Effect	Enhanced knowledge and skills on nutrition behaviors	Cooking balanced diet
		Savings
		Cultivating/kitchen garden
		Hygiene practices
	Perceived improved nutrition	Perceived improved nutritional status
		Positive mindset on nutrition
	Independence/empowerment	Improved financial literacy
		Economic strengthening
Challenges	Lack of awareness	Ignorance on the program
		Lack of interest in the program
	Negative attitude and beliefs	Religion
		Misconceptions/beliefs/customs/traditions
		Negative mindset (expecting only money)
	Economic constraints/poverty	Lack of food for demonstration
		Lack of money for buying food items
	Lack of support and time	Lack of spousal support
		Conflict with spouse
		Lack of caregiver at home
		Being busy looking for a job
		Busy at work
Limitation	Lack of inclusiveness for all social groups	Lack of inclusiveness
		Including only social classes 1 and 2
		Excluding social classes 3 and 4

The main themes are presented further followed by their subthemes. The findings are described with quotes generated from the FGDs and KIIs.

### Effect/benefits of the *Gikuriro* program (core theme 1)

#### Subtheme 1: enhanced knowledge and skills on nutrition

Most of the pregnant women and lactating mothers expressed their views on the perceived positive effect of the intervention and the benefits gained from the *Gikuriro* program. One of the main advantages mentioned frequently was enhanced knowledge and skills gained in cooking a balanced diet. They claimed that they did not know how to cook a balanced diet before the program was implemented. They expressed how lucky they were to have got the opportunity to gain knowledge and skills, especially on having a balanced diet from readily available food. Some of the views expressed were as follows:

“*Now we know how to cook a balanced diet with locally available items, which don’t require us to be rich. Before Gikuriro, we were ignorant, and we didn’t know how to cook a balanced diet. Now we have learned a lot from Gikuriro Program.*” (Nyabihu district, FGD_9_)

“*There were so many who were getting sick with malnutrition, not because they did not have sufficient food but because they didn’t know how to prepare a balanced diet.*” (Nyarugenge district, FGD_3_)

The same benefit was expressed by nutritionists who observed an improvement in skills on cooking a balanced diet quoted as follows:

“*Pregnant women and lactating mothers learned how to prepare a balanced diet. Before they used to think that a balanced diet meant eating meat but now, they know that it includes a variety of food*” (Kayonza district, Nutritionist KII_25_).

Another benefit was that *Gikuriro* had a great effect on the economic wellbeing of the women by enhancing their economic growth through saving groups.

“*Gikuriro project taught us how to save money, make money, borrow money, get into business, buy food, a farm and a lot of other things, I am satisfied, and I don't always have to ask for money from my husband.*” (Ngoma district, FGD_7_)

This improved the livelihoods of the households, especially for women because they stated that they no longer asked money from their husbands to buy food and other items, which was reinforced by a KII.

“*Gikuriro project has changed a lot for people by teaching them how to improve their income by creating saving groups and now they can buy vegetables and fruits from their savings without waiting from their husbands.*” (Kicukiro district, Nutritionist KII_2_)

Another subtheme category that emerged strongly was the improved knowledge and skills in kitchen garden and cultivation. Most participants claimed that the *Gikuriro* program had increased their knowledge and skills on how to promote increased agricultural productivity through kitchen gardens, which led to food security.

“*We have learned how to make different kitchen gardens which we did not know before Gikuriro came. Now we are planting different vegetables and fruits so that we have enough vegetables and fruits instead of buying them*.” (Ngoma district, FGD_7_).

In addition, rearing small domestic livestock improved significantly. For example, one key informant stated his views as follows:

“*Gikuriro encouraged people to start rearing small domestic animals to improve their livelihood. They have also been raising awareness about low-cost livestock breeding so that the family can earn a living from small livestock.*” (Kayonza district, Nutritionist KII_29_).

The study further revealed an increased awareness of hygiene practices, including maintaining safe and clean water, food and household utensils hygiene, and hand washing.

“*We used to struggle with hygiene and you could find households and parents with poor hygiene, but Gikuriro taught and alerted us about hygiene and sanitation practices such as how to keep water, food, household utensils clean; and how to wash our hands and encourage us to construct handwashing station around the latrine.*” (Ngoma district, FGD_8_).

“*Gikuriro has a special training program on the use of clean water, training of people called village social workers, and cleaning and sanitation to prevent disease from contamination.*” (Kicukiro district, CHO KII_14_).

#### Subtheme 2: perceived improved nutritional status and a positive mindset toward nutrition

Most participants voiced that the *Gikuriro* program led to perceived improved nutritional status among lactating mothers and pregnant women and their children aged <5 y. In addition, they testified that before the *Gikuriro* program was implemented, there were some children who were previously malnourished, but at the time of the study, they nourishment improved. This is how the discussants and key informants expressed their views:

“*Gikuriro program brought a change in terms of nutrition because we have learned how to cook a balanced diet, and now the children are well off and have no problems with malnutrition.*” (Kayonza district, FGD_6_).

“*Gikuriro really helped to reduce malnutrition significantly, for example, we used to have around 175 children with malnutrition in this sector but now after Gikuriro we have only about 10.*” (Ngoma district, CHO KII_42_).

Participants indicated that they had been expecting to get some money or other goods, but the *Gikuriro* project mainly taught them how to properly use what they have by changing their mindset positively. It was reported that people had changed their practices regarding the way they were feeding their children and themselves.

“*Gikuriro was a very good project, before people were used to being given money and they were wondering at the beginning what is the benefit, whether to get milk, rice, porridge, but the Gikuriro project was aimed at changing people's attitudes and training them to improve their lives based on what they already have.*” (Kicukiro district, Nutritionist KII_2_).

“*Before the Gikuriro program come, people were less likely to care about eating vegetables and fruits, but when the program came, people woke up and began to give importance to the inclusion of vegetables and fruits in their diet.*” (Kayonza district, Nutritionist KII_25_).

#### Subtheme 3: women empowerment/independence

The women spoke out highly that the *Gikuriro* program empowered them economically through savings because they claimed that they are no longer dependent on their husbands and were able to help their families.

“*Some of the saving groups they worked with made women less likely to expect anything from their husbands and be able to support their families.*” (Kicukiro district, CHO KII_5_).

Moreover, they were taught how to start small businesses and projects, such as soap making, food processing, sewing, making skirts, and weaving clothes, which in turn supported the women to become economically empowered.

“*Some women were isolated in their homes, but with the Gikuriro program, they are now in cooperatives. Some are producing soaps; others are doing other activities that allow them to be independent. They are no longer depending on their husbands.*” (Kayonza district, CHO KII_26_).

### Challenges of the *Gikuriro* program utilization (core theme 2)

#### Subtheme 1: lack of awareness on the importance of Gikuriro program

Under this subtheme, participants revealed that some women were ignorant and lacked awareness on the importance of *Gikuriro* program activities. They mentioned that the main challenges they faced were that some parents were not interested in *Gikuriro* project, which could be owing to poor knowledge of its importance.

“*The goal was to change people's attitudes, but some people were not available to join groups. They didn't think that these nutrition services were relevant to them.*” (Kayonza district, CHO KII_32_).

“*There is something I find that prevents pregnant and lactating mothers from attending the Gikuriro program which is ignorance, sometimes women do not understand the value of nutrition intervention projects.*” (Ngoma district, FGD_8_).

“*Some women initially were not interested but later they realized the importance of Gikuriro program and began to use the project.*” (Ngoma district, FGD_7_)

#### Subtheme 2: negative attitudes and beliefs

The findings revealed that some of the interventions may not have been culturally appropriate for all religions. For instance, some participants from certain beliefs/religions were not comfortable rearing animals, such as pigs or rabbits.

“*When Gikuriro proposed to give pig or rabbit, Adventists and Muslims refused though they were provided with options such as chickens.*” (Ngoma district, FGD_7_ and Kicukiro district, Nutritionist KII_11_).

Another barrier described was the misconception that some women thought the nutrition intervention was only for poor people or those having undernourished children, which led to low attendance at the beginning as some were ashamed to participate in the nutrition program.

“*Some had misconceptions that Gikuriro was for poor people or for those who have children with malnutrition, and they did not want to join the program at the beginning because they felt very poor and ashamed. This was the obstacle to justifying poor participation at the beginning.*” (Kayonza district, Nutritionist KII_31_).

“*There were some rich parents who felt like going to sit with someone who is poor is so disrespectful which prevented them from attending and most of the time they were the ones who were found with children who are malnourished, and that obstacle prevented them from attending Gikuriro programs which were good for their babies and themselves.*” (Ngoma district, FGD_8_)

It was observed that some of the participants were expecting to receive monetary benefits from the *Gikuriro* program rather than knowledge and skills.

“*Participation was not for everyone because there were those who gave up because they are not getting money and goods as many people were expecting to be given money or other things.*” (Kicukiro district, Nutritionist KII_2_).

However, contrary to this, other participants recommended that other projects should also not give money to participants for the projects to be sustainable.

“*My advice is for other projects not to give money to the people, instead, they should give them lasting things, because often the community gives them money to do something and use it sometimes without producing the same profit as it was intended.*” (Kicukiro district, Nutritionist KII_9_)

### Subtheme 3: economic constraints/poverty

Regarding the demonstrations on cooking a balanced diet, participants were required to bring the food items from home. This was because the main aim of the project was to help the community become self-sustainable and teach them to prepare a balanced diet with what they have. However, most informed that they did not have food and money to buy those food items, which led some to missing out, resulting in a low turnout of participation.

“*Some of the challenges pregnant women and lactating mothers face are lack of food because as it was time to go to the village kitchen, everyone had to bring food to come together to learn how to prepare a balanced diet and be ashamed of as they did not see what they had to do with others.*” (Nyabihu district, FGD_10_).

“*Women were asked to bring what they have at home to the nutrition education sessions but those poor women could not attend without bringing something, they thought that other women were going to laugh at them.*” (Kayonza district, Nutritionist KII_31_).

“*They are poor because of the lack of means/money and they wanted to be given food and prepare it, and someone would say,* ‘*I don't have it*.’” (Nyarugenge district, CHO KII_20_).

Although this mostly came off as a challenge, it was, however, seen as a positive effort to change the mindset of the community by other participants who expressed otherwise.

*Sometimes we tell them to bring food and some don’t have, but also the feeling that they had a responsibility to find food was useful, not like a project that comes and goes and brings food and then stops, which I see as a good thing that happened by Gikuriro program even though it was a challenge on the other hand that there were those who weren’t able to get what they were asked to bring for the cooking demonstration sessions.* (Kicukiro district, CHO KII_7_ and Kayonza district, CHO KII_28_).

#### Subtheme 4: lack of spousal support and time constraints

Furthermore, participants described that some pregnant women and lactating mothers lacked support, especially from their husbands to attend the *Gikuriro* program. Some reported that their husbands indicated that the program was for poor people. Another obstacle that women often faced was the lack of a caregiver at home to take care of other children who are not attending kindergarten and often missed out the programs. Lack of time, being busy looking for jobs, or working was another barrier to women attending the program.

“*Some people resist change. Here for instance, some men did not want their wives to attend Gikuriro activities thinking that those activities were for poor people*.” (Kayonza district, Nutritionist KII_31_).

“*Women have a lot of responsibilities in their homes, and sometimes they find themselves being busy at home including caring for children which may result in missing out Gikuriro programs, because she does not have someone to leave with the children.*” (Nyabihu district, FGD_10_).

“*The challenge was that some women were very poor and are busy looking for jobs or are always at work and failed to attend educational sessions on nutrition*.” (Kayonza district, CHO KII_28_).

### Limitations: lack of inclusiveness of the high social category (core theme 3)

Most participants felt that the *Gikuriro* program was excluding pregnant women and lactating mothers from the higher social categories because it was targeting those only from social categories 1 and 2. The socioeconomic status in Rwanda is assessed using poverty social category established by Minister for Local Government and Social Affair in 2015. These are 4 categories based on ascending income levels: category 1, extremely poor; category 2, poor; category 3, self-sustaining; and category 4, rich [17].

The program included mothers from higher social class only if their children were malnourished. The beneficiaries described that even women in high social classes may also lack the skills and knowledge on balanced diet, WASH, kitchen garden, and savings. Hence, the main recommendation for the success of such projects emanating from the beneficiaries is inclusion of all social categories.

“*The obstacles that existed are the Ubudehe social categories, because Gikuriro was for the first and second categories. A pregnant or breastfeeding mother in the third category who needed that help was not allowed into the program although they should be taught about nutrition.*” (Kayonza district, FGD_5_).

“*The only problem was that it was not accessible to all, it was only focused on women of the first and second wealth categories. Those in Ubudehe 3 and 4 were not allowed to participate though they wanted it*.” (Kicukiro district, Nutritionist KII_4_ and KII_13_; Kayonza district Nutritionist KII_29_).

“*It is also important to look at how targets in wealth categories 3 and 4 can be helped, as some of them have poor nutrition and find that this kind of help is not available to them as they do not know how to prepare a balanced diet.*” (Nyabihu district, CHO KII_47_).

“*My advice is that if there is another project, I wish it can give everyone the right to participate regardless of Ubudehe categories*.” (Kicukiro district, CHO KII_7_)

## Discussion

This qualitative study has demonstrated the various potential effects of the integrated nutrition-intervention package (*Gikuriro* program, in this case), such as enhanced knowledge and skills gained on cooking a balanced diet, kitchen gardening, saving and hygiene practices; perceived improved nutritional status; and a positive mindset regarding nutrition and women economic empowerment through social safety nets. However, some of the factors that were identified as challenges hampering the use of the integrated nutrition-intervention package were lack of awareness, negative attitudes/beliefs, economic constraints, and lack of husband support and time. In addition, the study found that one key limitation of the program, which was the lack of inclusiveness for all social groups.

Most of the study participants indicated that before *Gikuriro,* they neither knew how to cook a balanced diet with what they had nor how to make a kitchen garden for planting vegetables and fruits. This finding adds to the literature that communities exposed to integrated nutrition interventions are significantly more likely to gain knowledge about nutrition and are more likely to practice the behavior [18]. It is evident that enhanced cooking skills affects positively balanced diet by improving confidence in cooking and consumption of vegetables and fruits, and this is found to be beneficial among vulnerable and those of low socioeconomic status [19]. Furthermore, it is documented that cooking skills expose individuals to various new foods and facilitate compliance with the desired dietary guidelines for vegetables and fruit consumption [20, 21]. However, a number of intervention researches have revealed that translation of nutrition knowledge into practice of behaviors in nutrition programs is affected by availability, affordably, and accessibility of food items and time or resources constraints and effect of family members [18, [22], [23], [24], [25], [26]]. Therefore, nutrition-intervention implementers should consider these factors to translate the knowledge on nutrition into practice. Moreover, a follow-up study on nutrition behavior practice should be conducted for *Gikuriro* program to understand whether this knowledge has translated into practice and to explore the barriers of practice in Rwandan context.

Several studies in different countries have demonstrated that kitchen gardens improve food security of households [[27], [28], [29], [30]]. Moreover, it is evident from the literature that homestead food production interventions increase the production of micronutrient-rich foods, such as crops and fruits [10, 27]. Moreover, most of the participants from the study reported enhanced knowledge and skills on good hygiene practices and access to safe water and sanitation. There is evidence that these can reduce infectious diseases [31] because good nutrition requires safe water and sanitation [32].

Another effect of the nutrition-sensitive component mentioned highly in our study was the skills of group savings that led to women empowerment and economic independence to buy food items. This adds to the empirical evidence that women empowerment and autonomy improves nutritional status of mothers, their children, and other members of the household [[33], [34], [35]]. Literature shows that social safety nets increase income resilience, especially for the vulnerable groups, which leads to spending more on food and positive change in food consumption for pregnant women, lactating mothers, and children [10, 36] through improved access to resources [37]. However, women empowerment is a multidimensional domain where different domains, such as income, education, gender parity, agricultural production, and group membership empowerment, may influence the nutritional status through various mechanisms [26, 38]. In this study, the *Gikuriro* program had 2 domains: economic and agricultural production empowerment. Economic empowerment helps women to buy specific foods, and agricultural production empowerment is associated with feeding practices [33, 39].

All these improved knowledge and skills; in addition, a changed positive mindset regarding nutrition and women empowerment could have affected on the perceived improved nutritional status by addressing both immediate and underlying causes of malnutrition as highly expressed by many of the key informants and FGDs participants in the study. This is supported by quasi-experimental studies conducted by the same authors, which found that the *Gikuriro* intervention was associated with a significant reduction of low birth weight and maternal undernutrition [12, 13].

Despite the positive effect/benefits of the integrated nutrition-intervention program, this study identified some challenges and limitations to the implementation or use of services from the program. One of the subthemes of the challenges was a lack of awareness and negative beliefs/misconceptions. Under this subtheme, being ignorant and lacking knowledge on the importance of a balanced diet emerged as a key barrier. Respondents mentioned that some women were not interested and did not care about nutritious food even if they had a diversity of food. According to some participants, if they are satisfied, there is no problem. This is acknowledged by findings from another study that some individuals are resistant to change in dietary practices [40]. Similarly, maternal nutritional misconceptions and lack of knowledge are reported as core reasons for not attending nutrition-intervention programs among women [14, 41]. Therefore, utilization of nutrition interventions may not be influenced by the availability and accessibility but rather by a readiness to change behavior. This suggests that nutrition program implementers should also engage to increased awareness and nutrition behavior change.

Negative attitudes, such as the belief that nutrition interventions are only for the poor or malnourished, feeling ashamed to participate in the nutrition-intervention program because such programs are associated with having a malnourished child, and expecting only money from projects instead of knowledge and skills were reported to negatively affect participation in the integrated nutrition intervention. Although not very common, religious beliefs, which led to the refusal to rear animals, such as pigs, were also mentioned as barriers. Moreover, various studies have shown that community beliefs, cultural effects, and religious influences contribute to utilization of nutrition intervention [[42], [43], [44]]. These barriers may thwart pregnant women and lactating mothers from taking balanced and diversified nutritious food, which could affect their nutritional status and that of their babies [45]. Thus, integrated intervention programs should consider implementing culturally appropriate interventions for all beneficiaries in the community, with special considerations for groups with special belief-related barriers.

In this study, economic constraints were identified as a major barrier hampering attendance in the *Gikuriro* program. In the program, pregnant women and lactating mothers were required to bring food items to the village Kitchen School. The inability to obtain those foods was frequently mentioned by the study participants. Such a barrier may prevent pregnant women and lactating mothers from using nutrition interventions. Similarly, other studies reported that poverty and economic constraints were the key barriers to obtaining those foods and nutrition-intervention utilization among low social class families [[45], [46], [47], [48]]. Moreover, resource shortages, such as poverty and discrimination based on socioeconomic status in the community may hinder the uptake of nutrition interventions [49].

Furthermore, most of the participants identified lack of support and time as the main hurdles to using the nutrition-intervention program. Under this subtheme, lack of spousal support and lack of time owing to hefty workload at home or at work were key barriers preventing the women from attending the nutrition program. Similarly, studies have evidenced that heavy workload and poor husband support are barriers that limit pregnant women and lactating mothers in using integrated nutrition intervention and services [14, 50, 51]. As reported, in developing countries, husbands are influential in decisions related to health care service utilization [52]. Therefore, such programs should consider husbands' involvement in the interventions. Moreover, offering nutrition-intervention programs at the workplace and house level might be more feasible for women with limited time or spousal support.

### Strengths and limitations

The strengths of the study include using a big sample size of various community members, such as pregnant women, lactating women, nutritionists, and CHOs, involved in the integrated nutrition intervention. Another strength is the use of triangulation and confirmation from KIIs and FGDs by including different study groups to deeply explore the effect/benefits and challenges of using nutrition programs. However, attention should be given to the limitation of bias because of social desirability in which the study participants having been the beneficiaries and implementers of the program may over report the positive aspects of the program. To overcome this, to understand what the participants really felt about the barriers to the intervention, the investigators and facilitators clearly explained and emphasized that the purpose of the study was to inform future nutrition program implementation.

## Conclusion

The study has demonstrated several positive effects of an integrated nutrition-specific and nutrition-sensitive intervention package, such as promoting women empowerment, perceived improved nutritional status and enhanced knowledge/skills on cooking a balanced diet, kitchen garden, hygiene practices, and savings. Despite these benefits, there were some challenges identified that hindered pregnant women and lactating mothers from using the integrated nutrition intervention, including poverty/economic restrictions, lack of husband support and time, and lack of awareness and negative beliefs about the nutrition intervention. Finally, excluding social categories 3 and 4 was identified as a key limitation as emphasized by the study participants. Therefore, these challenges and limitations should be considered in future nutrition programs or interventions for more robust outcomes. Awareness campaigns on the misconceptions about nutrition interventions, involving husbands/men in the interventions, support for pregnant women and lactating mothers, and their economic development/empowerment should be enhanced.

## Author disclosures

MH, AGA, MU, MM, and CM, no conflicts of interest.

## Data Availability

The data of this study are available on request from the corresponding author.
